# Fecal microbiota transplantation inhibits colorectal cancer progression: Reversing intestinal microbial dysbiosis to enhance anti-cancer immune responses

**DOI:** 10.3389/fmicb.2023.1126808

**Published:** 2023-04-18

**Authors:** Hao Yu, Xing-Xiu Li, Xing Han, Bin-Xin Chen, Xing-Hua Zhang, Shan Gao, Dan-Qi Xu, Yao Wang, Zhan-Kui Gao, Lei Yu, Song-Ling Zhu, Li-Chen Yao, Gui-Rong Liu, Shu-Lin Liu, Xiao-Qin Mu

**Affiliations:** ^1^Genomics Research Center (Key Laboratory of Gut Microbiota and Pharmacogenomics of Heilongjiang Province), College of Pharmacy, Harbin Medical, University, Harbin, China; ^2^HMU-UCCSM Centre for Infection and Genomics, Harbin Medical University, Harbin, Heilongjiang, China; ^3^Translational Medicine Research and Cooperation Center of Northern China, Heilongjiang Academy of Medical Sciences, Harbin, Heilongjiang, China; ^4^Pathology Department, The Second Affiliated Hospital of Harbin Medical University, Harbin, Heilongjiang, China; ^5^Department of Colorectal Surgery, The Second Affiliated Hospital of Harbin Medical University, Harbin, Heilongjiang, China; ^6^Department of Microbiology, Immunology and Infectious Diseases, University of Calgary, Calgary, AB, Canada

**Keywords:** colorectal cancer, intestinal microbiota, fecal microbiota transplantation, inflammation, anti-cancer immune responses

## Abstract

Many lines of evidence demonstrate the associations of colorectal cancer (CRC) with intestinal microbial dysbiosis. Recent reports have suggested that maintaining the homeostasis of microbiota and host might be beneficial to CRC patients, but the underlying mechanisms remain unclear. In this study, we established a CRC mouse model of microbial dysbiosis and evaluated the effects of fecal microbiota transplantation (FMT) on CRC progression. Azomethane and dextran sodium sulfate were used to induce CRC and microbial dysbiosis in mice. Intestinal microbes from healthy mice were transferred to CRC mice by enema. The vastly disordered gut microbiota of CRC mice was largely reversed by FMT. Intestinal microbiota from normal mice effectively suppressed cancer progression as assessed by measuring the diameter and number of cancerous foci and significantly prolonged survival of the CRC mice. In the intestine of mice that had received FMT, there were massive infiltration of immune cells, including CD8^+^ T and CD49b^+^ NK, which is able to directly kill cancer cells. Moreover, the accumulation of immunosuppressive cells, Foxp3^+^ Treg cells, seen in the CRC mice was much reduced after FMT. Additionally, FMT regulated the expressions of inflammatory cytokines in CRC mice, including down-regulation of IL1a, IL6, IL12a, IL12b, IL17a, and elevation of IL10. These cytokines were positively correlated with *Azospirillum_sp._47_25*, *Clostridium_sensu_stricto_1*, the *E. coli* complex, *Akkermansia*, *Turicibacter*, and negatively correlated with *Muribaculum*, *Anaeroplasma*, *Candidatus_Arthromitus*, and *Candidatus Saccharimonas*. Furthermore, the repressed expressions of TGFb, STAT3 and elevated expressions of TNFa, IFNg, CXCR4 together promoted the anti-cancer efficacy. Their expressions were positively correlated with *Odoribacter*, Lachnospiraceae-UCG-006, *Desulfovibrio*, and negatively correlated with *Alloprevotella*, Ruminococcaceae UCG-014, *Ruminiclostridium*, Prevotellaceae UCG-001 and *Oscillibacter*. Our studies indicate that FMT inhibits the development of CRC by reversing gut microbial disorder, ameliorating excessive intestinal inflammation and cooperating with anti-cancer immune responses.

## 1. Introduction

Conventional methods for the treatment of colorectal cancer (CRC), such as surgical removal of the tissue and destruction or inhibition of the cancer cells by chemo- or radio-therapies, may prolong the life of the patient but in the meantime may inevitably damage normal tissues and organs, especially the immune system, often making the patient even more vulnerable to infections and new malignancies. Additionally, chemo- and radio-therapies often induce resistance. Such situations, coupled with the steadily growing morbidity, call for novel therapeutic strategies for the treatment of CRC.

Over the past decades, increasing evidence indicates the existence of microbiota-host homeostasis in the human gut, which cooperates with the immune system to defend against diseases, especially infections and malignancies. Disturbance to such homeostasis by physicochemical or biological factors, e.g., abusive use of antimicrobials or unhealthy diets as well as natural events such as infections, has been associated with cancers and many other disorders (Ansaldo et al., 2021; Pan et al., 2021; Sepich-Poore et al., 2021). As a result, research interest is growing in fecal microbiota transplantation (FMT) to recover the microbiota-host homeostasis for the treatment of cancer. However, how the disturbed or recovered intestinal microbiota influence the development of CRC remains unclear.

The human gut is colonized by thousands of bacterial species with an average mass of 1.5 kg and more than 10^14^ cells. These diverse microbes maintain a dynamic balance and play important roles in the stability of the microbiota-host homeostasis. However, phylogenetic composition and population structure of the intestinal microbiota significantly differ between CRC patients and healthy people (Cheng et al., 2020). In CRC patients, some bacteria showed increased abundance, e.g., *Porphyromonas*, *Enterococcus*, *Streptococcus*, or *Peptostreptococcus*; conversely, some bacteria showed decreased abundance, such as *Roseburia* and other butyrate-producing bacteria in the family Lachnospiraceae (Alhinai et al., 2019). Among these microbiota, oncogenic bacteria have been reported in CRC patients. Bacteria of *Streptococcus bovis* biotype I, for instance, are known to be associated with colorectal adenoma and CRC: they may invade intestinal epithelial cells and in the meantime escape the surveillance of the immune system (Agnes et al., 2021). Other reported CRC-associated bacteria include *E. coli* harboring the *pks* gene island (Liu et al., 2021), *Bacteroides fragilis* secreting toxin to cause DNA damage (Alhinai et al., 2019; Cao et al., 2021). On the other hand, intestinal bacteria that play protective roles against CRC have also been reported, such as *Lactobacillus plantarum YYC-3* (Yue et al., 2020) and *Lactobacillus acidophilus* (Li S. et al., 2019), which produce beneficial metabolites that the host does not produce. In addition, chemotherapy (5-fluorouracil/oxaliplatin) markedly increases the ratio of relative abundance of the Firmicutes/Bacteroidetes, inducing greater disturbance of intestinal microbiota (Chang et al., 2020). Although the gut microbiota of patients who had a partial response for targeted therapy (bevacizumab/cetuximab) exhibits significantly higher α-diversity than that of the progressive disease group, the overall intestinal microbiota is still disordered (Chen et al., 2022). Alteration in the intestinal microbiota affects the metabolic pathway of glycerophospholipid, thereby regulating the therapeutic potential of the immunotherapy (PD-1 antibody) in MSS-type CRC tumor-bearing mice (Xu et al., 2020). These findings together strongly indicate the importance of a healthy intestinal microbiota in defense and prevention against malignancies such as CRC.

We chemically induced gut microbial dysbiosis and CRC and conducted FMT from healthy mice to CRC mice to assess the associations of gut microbial changes with CRC development and found that FMT alleviated the disease severity and significantly increased the survival rate of the CRC mice by reversing gut microbial dysbiosis and regulating the level of intestinal inflammation and immune responses.

## 2. Materials and methods

### 2.1. The CRC mouse model

**Figure 1:**
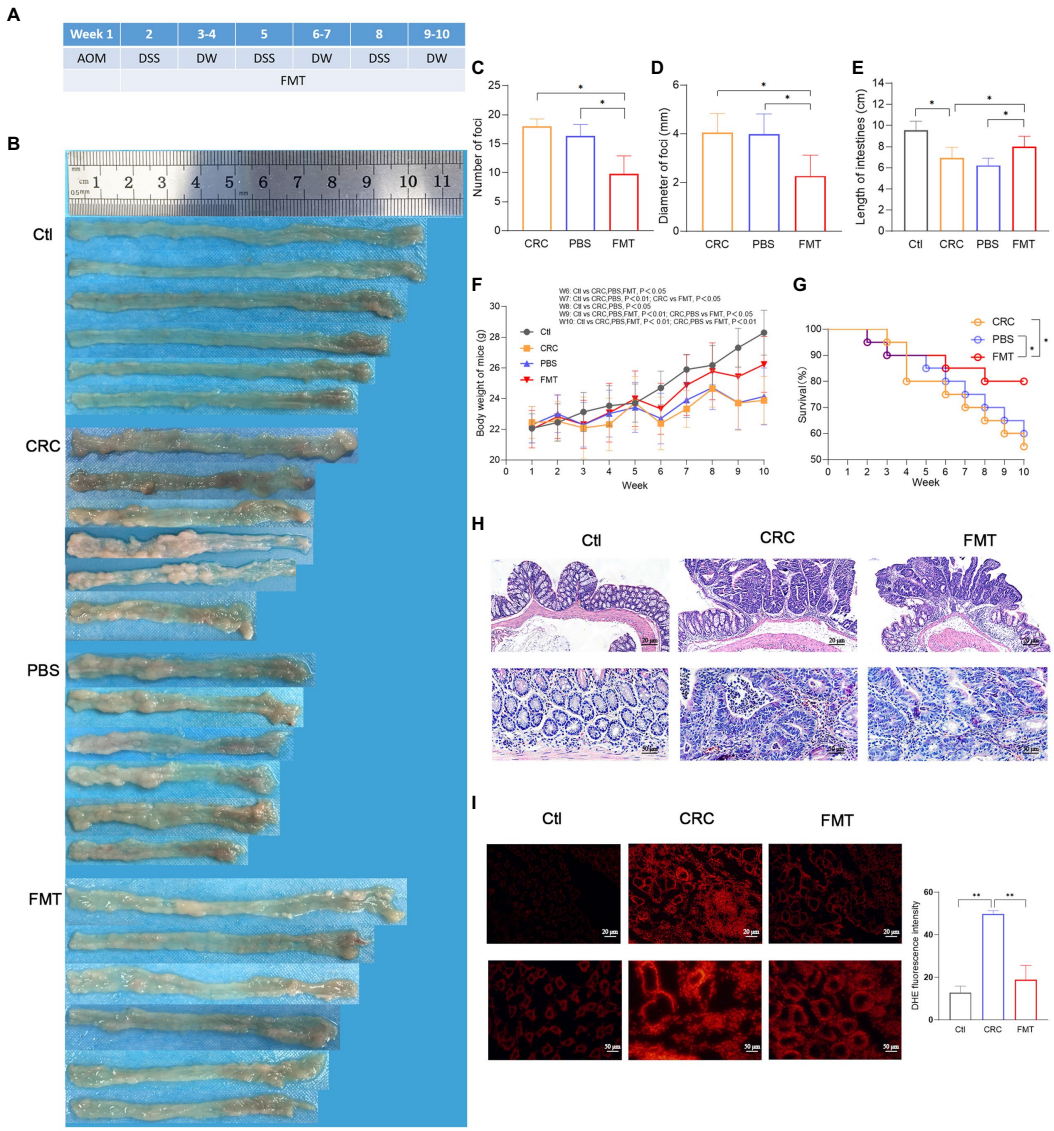
Suppressive effect of FMT on CRC progression. **(A)** Timeline of experimental procedure. **(B)** Pictures of intestinal tissue from each experimental group, including normal control mice (Ctl), mouse model of colorectal cancer (CRC), CRC mice treatment with PBS as negative control (PBS), and CRC mice receiving fecal microbiota transplantation (FMT). **(C–E)** The average number, diameter of cancerous foci and the length of intestinal tissues in each group. *p < 0.05 (Ctl, *n* = 20; CRC, *n* = 11; PBS, *n* = 12; FMT, *n* = 16). **(F,G)** The average body weight and survival rate of mice in each week. *p < 0.05 (Ctl, *n* = 20; CRC, *n* = 11; PBS, *n* = 12; FMT, *n* = 16). **(H)** HE staining of intestinal tissue. **(I)** Production of superoxide in the intestine indicated by the intensity of red fluorescence. **p < 0.01, *n* = 3.

Male Balb/c mice aged 8 weeks were purchased from Beijing Vital River Lab (Beijing, China) and acclimated for 1 week in SPF laboratory animal center (Suzuki et al., 2006). In week 1 of the experiment, the mice were intraperitoneally injected with the carcinogen azoxymethane (AOM, 10 mg/kg, Sigma). In week 2, the mice were free to drink water containing the proinflammatory agent dextran sodium sulfate (DSS, 2.5%, MP Biomedicals). In week 3 and week 4, the mice were given drinking water without DSS. Such treatment from week 2 to week 4 was repeated for two additional cycles (Figure 1A) to establish the CRC mouse model as described previously (Neufert et al., 2007). The mice were randomly allocated into three experimental groups, 4 mice per cage, 5 cages in each group. The study was approved by the Ethics Committee of the Harbin Medical University (HMUIRB20180013).

### 2.2. Fecal microbiota transplantation

Fecal samples for FMT were freshly collected from healthy, untreated and age-matched Balb/c donor mice. The collected fecal samples were thoroughly mixed in PBS (200 mg/ml) and centrifuged for 1 min at 1,500 rpm. The supernatant was then separated for transplantation. Each recipient mouse received 1 ml of the microbial solution by enema once every 3 days for 9 weeks. PBS enema of equal volume was performed as a negative control for FMT.

### 2.3. HE staining

Paraffin sections of colorectal tissues were stained with hematoxylin for 2 min, and then with eosin for 1 min. After dehydrating and hyalinizing, respectively, with ethanol and xylene, the tissues were sealed with neutral resin, and then imaged.

### 2.4. Detection of superoxide by dihydroethidium staining

The colorectal tissues freshly dissected in RPMI 1640 medium (HyClone) were stained in dark with 30 μM dihydroethidium (DHE, Invitrogen) for 5 min at room temperature. The tissues were then washed and immediately imaged by laser scanning confocal microscope at 420/590 nm. DHE exhibited blue-fluorescence in the cytosol. Once it was oxidized and intercalated with the DNA, fluorescence in the cell nucleus would be turned into a bright fluorescent red color. The relative red fluorescence intensity was analyzed by fluorescence microscope analysis system (Olympus).

### 2.5. High throughput sequencing and bioinformatics analysis of the full-length 16S rDNA of intestinal microbiota

The mice were kept for 1 week without any treatment after the completion of the whole FMT plan in order to exclude the negative impact of residual transplant fluid on the analysis of the reconstructed intestinal flora. Then, the mice were transferred to a clean cage for the collection of fresh fecal samples. Two tubes of feces were collected per cage, and 5–6 tubes of feces from different cages in same experimental group were used for high-throughput sequencing of intestinal microbiota (GENEWIZ, Suzhou, China). The PacBio sequencing platform was employed to obtain the full-length of 16S rDNA for its advantages of longer length of gene reads and higher accuracy. Accordingly, the data for analysis included five or six replicates in each experimental group and were submitted to NCBI (PRJNA606977). Microbial diversity was analyzed based on the representative OTU sequence with 97% similarity.

### 2.6. Immunohistochemical detection of immune cells

Paraffin sections were dewaxed in xylene and rehydrated in ethanol, respectively. Antigen retrieval was performed in a microwave oven for 2 min at high power and then for 10 min at low power, followed by cooling down to room temperature. The activity of endogenous peroxidase was quenched with 3% H_2_O_2_ for 10 min. Tissue blocking was performed with goat serum for 1 h at room temperature. Tissue sections were then incubated at 4°C over-night with primary antibodies for T lymphocytes (CD3, ARG52744, Arigo), helper T lymphocytes (CD4, ab183685, Abcam), cytotoxic T lymphocytes (CD8, ab217344, Abcam), Treg lymphocytes (FOXP3, GB11093, Servicebio), natural killer cell (CD49b, AGR57601), or macrophages (F4/80, ARG22476, Arigo). The sample were then incubated with horseradish peroxidase labeled secondary antibody (1: 500) for 1 h at 37°C. Color development was performed using the DAB peroxidase substrate kit (ZSGB-BIO) according to the manufacturer’s instruction. The sections were finally dehydrated in ethanol and xylene, and sealed with neutral resin. The image signal recorded by microscope was converted into digital signal by Image J to analyze the distribution and staining intensity of positive markers.

### 2.7. Mouse cancer inflammation and immunity crosstalk PCR Array

In order to explore the effect of intestinal microbiota on the intestinal inflammatory responses in CRC mice, we detected the expressions of genes coding for the cellular mediators of inflammation and immunity by the Mouse Cancer Inflammation & Immunity Crosstalk RT^2^ Profiler PCR Array (QIAGEN). The genes are as follows: *Ackr3*, *Aicda*, *Bcl2*, *Bcl2l1*, *Ccl2*, *Ccl20*, *Ccl22*, *Ccl28*, *Ccl4*, *Ccl5*, *Ccr1*, *Ccr10*, *Ccr2*, *Ccr4*, *Ccr5*, *Ccr7*, *Ccr9*, *Cd274*, *Csf1*, *Csf2*, *Csf3*, *Ctla4*, *Cxcl1*, *Cxcl10*, *Cxcl11*, *Cxcl12*, *Cxcl2*, *Cxcl5*, *Cxcl9*, *Cxcr1*, *Cxcr2*, *Cxcr3*, *Cxcr4*, *Cxcr5*, *Egf*, *Egfr*, *Fasl*, *Foxp3*, *Gbp2b Gzma*, *Gzmb H2-D1*, *H2-K1*, *Hif1a*, *Ido1*, *Ifng*, *Igf1*, *Il10*, *Il12a*, *Il12b*, *Il13*, *Il15*, *Il17a*, *Il1a*, *Il1b*, *Il1r1*, *Il2*, *Il22*, *Il23a*, *Il4*, *Il5*, *Il6*, *Irf1*, *Kitl*, *Mif*, *Myc*, *Myd88*, *Nfkb1*, *Nos2*, *Pdcd1*, *Ptgs2*, *Spp1*, *Stat1*, *Stat3*, *Tgfb1*, *Tlr2*, *Tlr3*, *Tlr4*, *Tlr7*, *Tlr9*, *Tnf*, *Tnfsf10*, *Trp53*, and *Vegfa*.

### 2.8. Western blot

Tissue proteins from each sample were denatured in 5× SDS-PAGE loading buffer and electrophoresis was performed on 12% tris-glysine agarose gels. The separated proteins were transferred to nitrocellulose membrane followed by blocking with 5% non-fat milk for 2 h at room temperature. The membrane was then incubated at 4°C over-night using primary antibodies for IFNg (ARG21502, Arigo), IL1a (ARG56667, Arigo), IL6 (ARG56625, Arigo), IL12a (bs-0767R, Bioss), IL12b (bs-10641R, Bioss), IL17a (ARG55256, Arigo), TNFa (ARG10158, Arigo), IL10 (ARG21475, Arigo), TGFb (ARG10002, Arigo), CXCR4 (ARG54674, Arigo), STAT3 (ARG54150, Arigo) and Tubulin (AC015, Abclonal). This was followed by incubation with horseradish peroxidase labeled secondary antibody (AS028, AS014, Abclonal; bs-0296G-HRP, Bioss) for 2 h at room temperature. Band intensity was measured by Tanon-5200 and Tanon MP.

### 2.9. Statistical analysis

Statistical analysis was performed by the Student–Newman–Keuls-q test and Log-rank test, and the statistical comparisons were performed by Graphpad Prism v5. All data were expressed as mean ± SD. A two-tailed value of *p* < 0.05 was taken to indicate statistical significance.

## 3. Results

### 3.1. FMT inhibits the enlargement of cancerous foci and prolongs survival of CRC mice

The schedule of making a colorectal cancer mouse model and performing intestinal microbiota transplantation are shown in Figure 1A. A large number of cancerous foci were observed in the intestine of the CRC mice, with the average number of 17.6 and the average diameter of 4.4 mm, whereas in CRC mice that accepted FMT, the average number of the foci was reduced to 9.8 with the average diameter of 2.3 mm (Figures 1B–D). The CRC mice showed the typical characteristic of intestinal tissue shortening induce by inflammatory adhesion and epithelial hyperplasia, while FMT significantly inhibited the intestinal tissue shortening (Figure 1E). Disordered intestinal crypts with cell dysplasia and hyperplasia could be clearly observed in the CRC mice with or without FMT (Figure 1F). The loss of body weight is a significant feature of CRC. The results showed that the body weight of CRC mice was significantly less than that of mice in the normal control group at the same feeding time point, with a difference of as much as 4.5 g at the end of the experiment. However, the difference was reduced by the treatment of FMT (Figure 1G). The survival of CRC mice treated with FMT prolonged significantly from the fifth week. At the end of the experiment, this value was elevated from 55% (11/20) to 80% (16/20) by the treatment of FMT (Figure 1H). A large amount of superoxide is produced during the rapid division of cancer cells, which in turn greatly facilitates carcinogenesis. In CRC mice, the release of superoxide was significantly increased in the intestine in comparison with the mice in the control group, as indicated by the bright DHE fluorescence around the crypts, whereas the release was significantly reduced in CRC mice accepted FMT, as shown by the dim red fluorescence (Figure 1I).

### 3.2. FMT reverses the dysbiosis of intestinal microbiota in CRC mice

**Figure 2:**
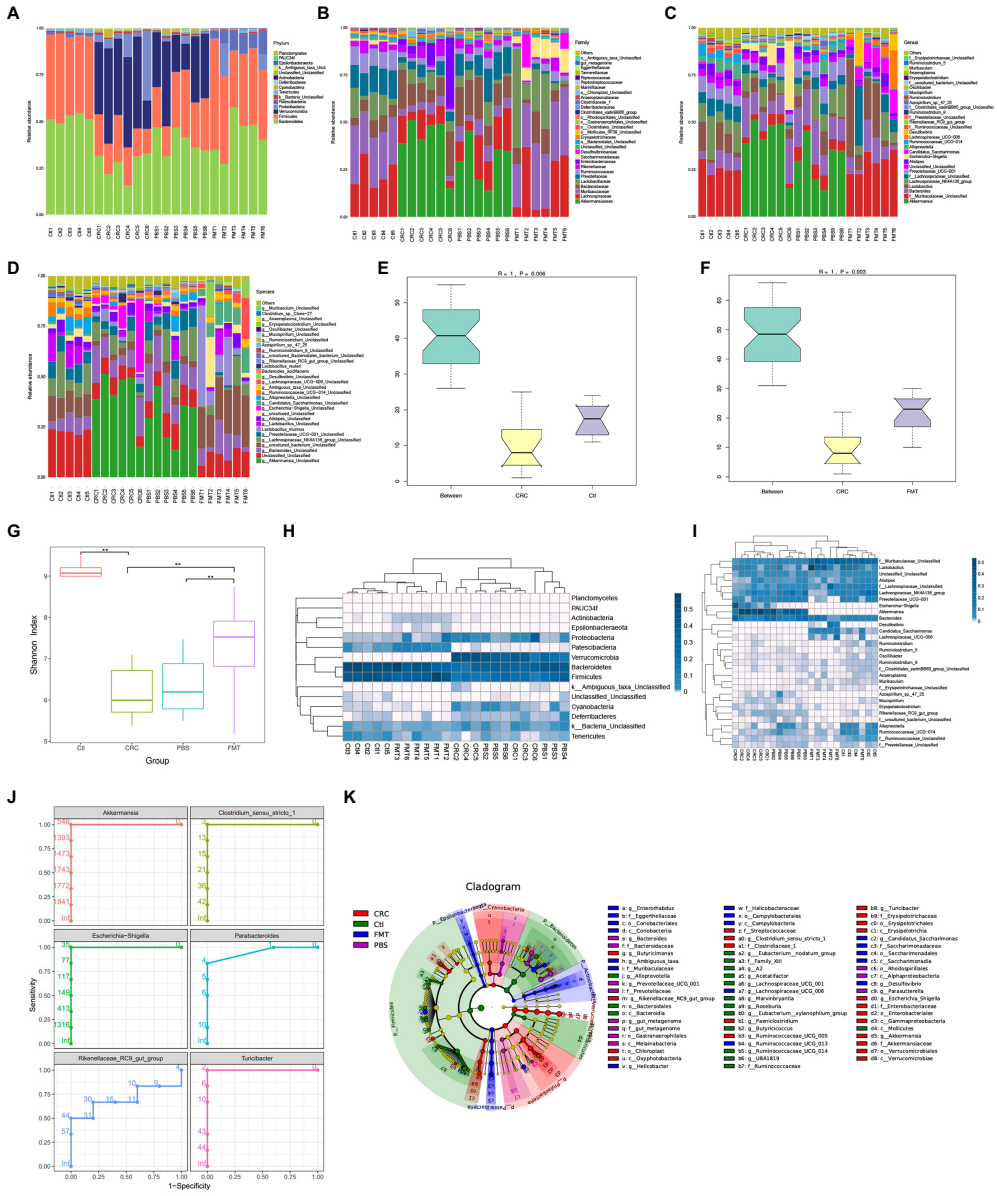
FMT restored intestinal microbial abundance and diversity of CRC mice. The distribution of intestinal microbiota in different classification, including phylum **(A)**, family **(B)**, genus **(C)** and species **(D)**. **(E,F)** Anosim analysis between samples. The R value towards 1 indicates that the difference between groups is much more than that within groups. *p* < 0.05 (Ctl, *n* = 5; CRC, *n* = 6; FMT, *n* = 6). **(G)** Shannon index analysis reflects the diversity of intestinal microbiota, including both microbial richness and evenness. **p < 0.01, (Ctl, *n* = 5; PBS, *n* = 6; CRC, *n* = 6; FMT, *n* = 6). **(H,I)** the weighted unifrac analysis at the levels of phylum and genus, respectively. **(J)** Receive operating characteristic curve reflects continuous variables of sensitivity and specificity. The area value under the curve prone to 1, indicating that the intestinal bacteria could be considered as marker of CRC mice. **(K)** LefSe analysis identifies representative bacteria in each group.

A total of 291,444 gene reads were obtained, with an average of 12,301 reads and 3,589 OTUs per sample after being processed by the quality filters. The intestinal microbiota was categorized to 15 phyla, 23 classes, 31 orders, 60 families, 148 genera and 203 species. In comparison with normal control mice, the abundance of intestinal microbiota was significantly altered in CRC mice at the level of phyla, family, genus and species, respectively (Figures 2A–D). Anosim analysis showed that the microbial diversity was also statistically different between mice of the CRC and control groups (Figures 2E,F). By the treatment with FMT, the microbial abundance and diversity of CRC mice were both significantly improved as shown by the shannon index analysis (Figure 2G). The phylogenetic structure of the microbiota in CRC mice was also significantly different from that in normal mice, but FMT altered the microbial structure in CRC mice toward that of the normal control mice (Figures 2H,I). Compared with CRC mice, PBS treated mice showed no significant improvement in the abundance, diversity and phylogenetic structure of intestinal microbiota.

Based on 16 s rDNA sequencing data, the classification of intestinal bacteria is generally accurate to the genus level. At the genus level, the dominant bacteria in mice of the normal control group included those in *Lactobacillus* (9.19%), *Alistipes* (3.69%), *Alloprevotella* (4.99%), *Ruminiclostridium* (1.50%), *Saccharimonas* (1.11%), *Oscillibacter* (0.55%), *Roseburia* (0.47%), *Muribaculum* (0.45%), *Anaeroplasma* (0.44%), *Arthromitus* (0.27%), and genera of Muribaculaceae (25.53%), Lachnospiraceae (20.52%), Prevotellaceae (7.51%), Ruminococcaceae (4.55%) and Erysipelotrichaceae (0.18%), which were all significantly decreased in the CRC mice. After FMT, most of these taxa increased and approached the normal levels. In contrast, some bacteria accounted for large proportions of the gut microbiota in CRC mice, including *Akkermansia* (40.72%), *Bacteroides* (13.83%), the *E. coli* complex (9.79%), *Clostridium* (0.60%), *Turicibacter* (0.54%), *Azospirillum* (0.29%), *Paeniclostridium* (0.20%), *Mucispirillum* (0.16%), *Parabacteroides* (0.15%), *Erysipelatoclostridium* (0.14%), and genera of Rikenellaceae (0.41%), some of which could be regarded as the marker of CRC mice through the analysis of receiver operating characteristic curve, that were *Akkermansia*, *Escherichia-Shigella*, Rikenellaceae RC9-gut-group, *Clostridium_*sensu_stricto_1, *Parabacteroides* and *Turicibacter* (Figures 2C,J). The proportions of these dominant taxa were significantly reduced after the treatment of FMT. Taking the advantage of detecting longer gene sequences by PacBio platform for more accurately classification of bacteria at species level, we obtained 90 species in mice of the control group, including *Lactobacillus murinus* (0.24%), *Bacteroides acidifaciens* (0.12%), *Lactobacillus reuter* (0.77%), *Azospirillum* sp.-47–25 (0.02%), and *Clostridium* sp.-Clone-27 (0.42%), with 113 additional taxa (OTUs) remaining to be classified for a formal taxonomic status at the species level since strains containing these gene sequences have not been included in the current database. Most notably, bacteria that are not colonized in normal mice and FMT treated mice, were detected in CRC mice such as *Clostridium_perfringens* (0.20%), *Escherichia_coli_*DEC14A (0.06%), *Odoribacter_*sp._N54.MGS-14 (0.02%), and *Escherichia_coli_*TOP291 (0.02%; Figure 2D).

Although the microbial disorder of CRC mice has been largely reversed, some intestinal bacteria are still difficult to colonize in the intestine through FMT, that were *Alloprevotella*, Ruminococcaceae*_UCG-014*, Lachnospiraceae*_UCG-001*, *Marvinbryantia*, and *Eubacterium-*xylanophilum-group as revealed by LefSe analysis (Figure 2K). It is necessary to isolate and culture these bacteria for multiple transplantation to increase the probability of their colonization.

### 3.3. FMT influences the recruitment of immune cells in the intestine and enhanced immune responses against CRC

**Figure 3:**
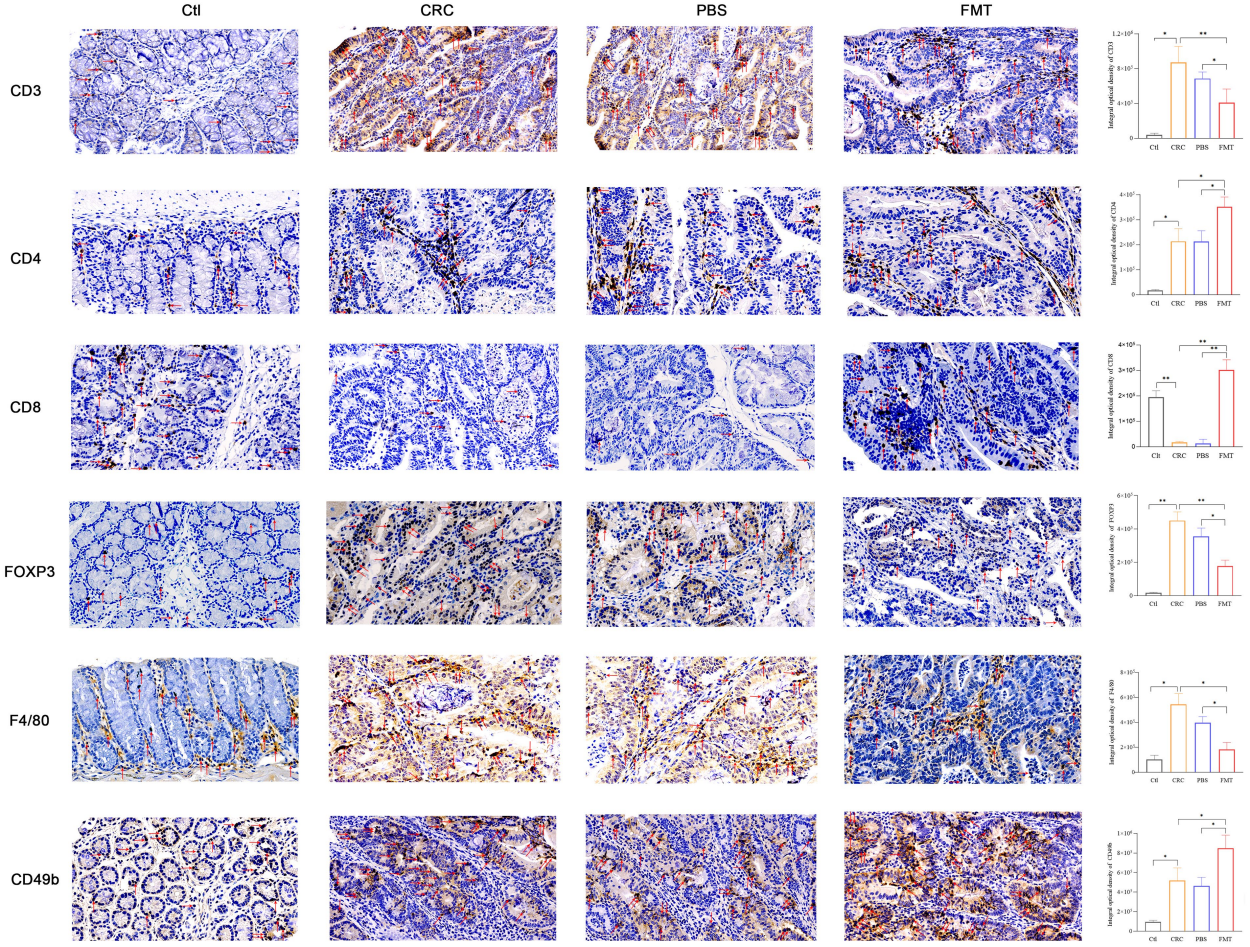
FMT enhanced immune cells mediated anti-cancer efficacy. The cytomembrane and cytoplasm of positive cells was stain brown. Microscopic photography scanning system (Zeiss) was used to record the images of intestinal tissue section of mice in Ctl, CRC, PBS and FMT groups, respectively. The average optical density of all immunohistochemically stained specimens was analyzed by Image J system. *p < 0.05, **p < 0.01, *n* = 3.

Intestinal immune cells are located at intestinal epithelium that separates the body from outside environment as an impermeable barrier. Due to this specific location, these cells directly contact enterocytes and are in proximity to antigens in the gut lumen. The dysbiosis of intestinal microbiota may trigger immune disorder *via* abnormal recruitment of immune cells in the intestine. In CRC mice with intestinal microbial dysbiosis, we found that the infiltration of nearly all T cells were significantly increased as shown by CD3 labeled positive cells, indicating robust immune responses. These increased tumor-infiltrating lymphocytes include CD4^+^ T cells, Foxp3^+^ Treg cells. In addition to adaptive immunity, innate immunity was also involved, manifested as elevated accumulation of F4/80^+^ macrophages and CD49b^+^ NK cells. Interestingly, the enrichment of CD8^+^ T cells and much more aggregation of CD4^+^ T cells, CD49b^+^ NK cells were observed in CRC mice accepted with FMT, as well as reduced population of Foxp3^+^ Treg cells, F4/80^+^ macrophages, indicating the augmentation of anti-cancer efficacy (Figure 3).

### 3.4. FMT influences the expressions of cytokines within CRC microenvironment to enhance immune responses against CRC

**Figure 4:**
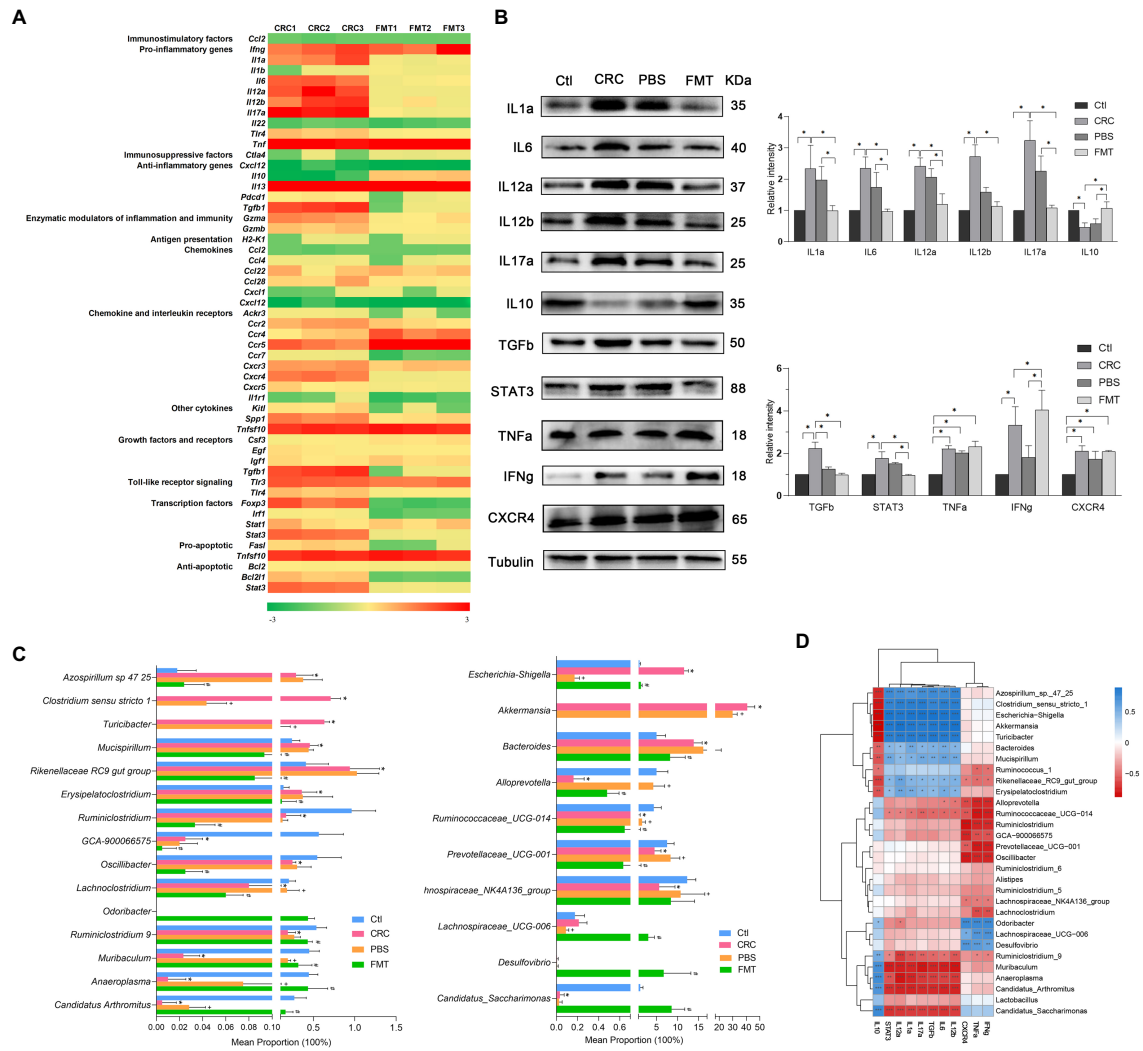
FMT regulated the expressions of cytokines in CRC microenvironment to enhance anti-inflammatory and anti-cancer efficacy. **(A)** PCR array analysis of cytokines within CRC microenvironment. **(B)** Protein images of differentially expressed cytokines screened by gene chip; the relative protein expressions of inflammatory cytokines IL1a, IL6, IL12a, IL12b, IL17a, IL10 and anti-cancer immunoregulatory factors TGFb, STAT3, TNFa, IFNg and CXCR4. *p < 0.05, *n* = 3. **(C)** The abundance of intestinal microbe related to inflammatory cytokines and anti-cancer immune molecules. *p < 0.05, Ctl vs. CRC, ^+^*p* < 0.05, CRC *vs* PBS, ^#^*p* < 0.05, PBS *vs* FMT, *n* = 6. **(D)** Correlation analysis between intestinal microbiota and cytokines. *p < 0.05, **p < 0.01, ***p < 0.001, *n* = 3.

On intestinal tumorigenesis, intestinal microbe translocate across altered epithelium and stimulate the recruitment of immune cells to release cytokines. These molecules within CRC microenvironment are sensitive indicators of microbiota-host homeostasis. In this study, dozens of genes enriched in the intestine of CRC mice were identified, including pro-inflammatory factors *Ifng*, *Il1a*, *Il6*, *Il12a*, *Il12b*, *Il17a*, and *Tnf*; immunosuppressive factors *Il13* and *Tgfb1*; enzymatic modulators *Gzma*; chemokine and interleukin receptors *Ccr5*, *Cxcr3*, and *Cxcr4*; other cytokines *Spp1* and *Tnfsf10*; toll-like receptor *Tlr3*; transcription factors *Foxp3*, and *Stat3*. By the treatment with FMT, the gene expressions of some molecules were altered significantly, such as suppressed expressions of *Il1a*, *Il6*, *Il12a*, *Il12b*, *Il17a*, *Tgfb1*, *Cxcr4*, *Spp1*, *Foxp3*, *Stat3*, and increased expressions of *Tnf*, *Il10*, and *Ccr5* (Figure 4A).

The differential expressions of these genes were further confirmed at protein level. In comparison with the normal control mice, the protein expressions of IL1a, IL6, IL12a, IL12b, IL17a, TGFb, STAT3, TNFa, IFNg and CXCR4 were significantly elevated in CRC mice, resulting in severe inflammatory responses manifested as hematochezia and lethargy, as well as activation of oncogene transcription. Interestingly, in mice received FMT, the expressions of these proteins were significantly altered, including down-regulation of pro-inflammatory factors IL1a, IL6, IL12a, IL12b, IL17a and up-regulation of anti-inflammatory factor IL10, contributing to obvious suppression of intestinal inflammation. Furthermore, the repressed expressions of growth factor TGFb, transcription factor STAT3 and elevated expressions of TNFa, IFNg, CXCR4 together promoted the anti-cancer efficacy (Figure 4B).

The expressions of pro-inflammatory factors IL1a, IL6, IL12a, IL12b, IL17a, growth factor TGFb and transcription factor STAT3 were positively correlated with *Azospirillum* sp.-47–25, *Clostridium sensu stricto*-1, *Escherichia-Shigella*, *Akkermansia*, *Turicibacter*, *Bacteroides*, *Mucispirillum*, Rikenellaceae RC9_gut_group and *Erysipelatoclostridium*. It was indicated that the expressions of TGFb and STAT3 were affected by intestinal bacteria related to inflammation and possibly the interaction between inflammatory environment and these microbe may promote the carcinogenic process. Therefore, these microbe were of great potential to serve as anti-inflammation and anti-cancer targets. In contrast, their expressions were negatively correlated with Ruminococcaceae UCG-014, *Ruminiclostridium*_9, *Muribaculum*, *Anaeroplasma*, *Candidatus*_*Arthromitus* and *Candidatus*_*Saccharimonas*, which show the potential to be developed as microecological agents for the treatment of colitis and CRC. In addition, the abundance of *Muribaculum* and Candidatus_*Arthromitus* in PBS treated mice were relatively higher than that in CRC mice, which are easy to colonize in the intestine, and PBS washing provide them more space for colonization. Their increased colonization may be the reason for the decreased protein expressions of IL12b and TGFb in PBS treated mice. Moreover, the expressions of cytokines which were able to directly kill cancer cells such as TNFa, IFNg and CXCR4 were positively correlated with *Odoribacter*, Lachnospiraceae UCG-006, *Desulfovibrio*, and negatively correlated with *Alloprevotella*, *Ruminiclostridium*, Prevotellaceae UCG-001, *Oscillibacter* and Lachnospiraceae NK4A136. These intestinal bacteria also show the potential to be developed as microecological agents and anti-cancer targets (Figures 4C,D).

## 4. Discussion

Growing evidence supports the pivotal role of intestinal microbiome as key regulatory elements in host’s physiological and pathological status. We have reported the associations of malignant diseases with gut microbiome dysbiosis (Zhuang et al., 2019), correlations of low *E. coli* genetic diversity with malignancies (Tang et al., 2020), and successful treatment of intestinal malignancy by ameliorating the gut microbiota with dietary lignans (Wu H. et al., 2021). Here we experimentally validated the previous findings and provided experimental evidence showing the potent activities of normal gut microbiota against CRC. Our results demonstrates that such anti-cancer activities are based on the cooperation between gut microbes and host immune responses.

Intestinal microbe such as *Lactobacillus, Alistipes, Saccharimonas, Alloprevotella, Ruminiclostridium, Oscillibacter, Anaeroplasma, Muribaculum, Arthromitus, Roseburia*, genera of Muribaculaceae, Lachnospiraceae, Prevotellaceae, Ruminococcaceae, and Erysipelotrichaceae, accounted for predominant proportions in normal mice of this study. It is reported that *Lactobacillus* reduces the level of inflammation, DNA damage and tumor load through down-regulation of polyamine components primed by the production of antioxidant glutathione and anti-angiogenic factors (Kim et al., 2021). In addition, *Lactobacillus* delays tumor initiation by increasing infiltration of NK cells, CD8^+^ T cells and production of IFNg in cancer microenvironme (Lau et al., 2021; Yang et al., 2021). *Lactobacillus* also have been shown to exert a therapeutic effect on liver injury by overproduction of anti-inflammatory factor IL10 in serum (Lv et al., 2014). Overexpressions of chemokine receptors associates with infiltration of T cell populations. In this study, we found that chemokine receptor CXCR4 were over expressed in CRC mice received FMT, which facilitate the binding to CXCL12 that generally expressed in cytotoxic CD8^+^ T cells, resulting in the significantly infiltration of CD8^+^ T cells. Remarkably, the chemokine receptor is less expressed *in vitro* cultured CRC cell lines than primary cancer cells, indicating that chemokine receptor expression in cancer cells may be stimulated by intestinal microenvironment factors absent *in vitro*, such as intestinal microbe (Cremonesi et al., 2018). Massive infiltration of CD8^+^ T cells is accompanied by the decreased abundance of *Ruminiclostridium*, Prevotellaceae_UCG_001, as well as enrichment of Lachnospiraceae, which was also indicated in our research results (Yu et al., 2020; Jing et al., 2021). Furthermore, introduction of *Helicobacter hepaticus* increases tumor infiltration by cytotoxic lymphocytes and the anti-tumor immunity is dependent upon CD4^+^ T cells and NK cells (Overacre-Delgoffe et al., 2021). Short chain fatty acids, produced by Lachnospiraceae and *Roseburia*, not only maintain the integrity of intestinal wall and directly induce apoptosis of cancer cells, but also act on the FFAR2 receptor in the intestine to influence the secretion of chemokines, thus altering the distribution of immune cells or directly acting on T-lymphocytes to regulate their differentiation, recruitment, activation and cytokine secretion (Koh et al., 2016; Makki et al., 2018). Bacteria of the Lachnospiraceae also strengthen the intestinal barrier by enhancing the tight junctions of intestinal epithelial cells and increasing the production of mucin (Verstrepen et al., 2021). Suppressive JAK2/STAT3 signaling was involved in limiting S100a8/9 production in colonic neutrophils to maintaining gut microbial ecology and colon homeostasis (Loh et al., 2021). Additionally, some bacteria in the Ruminococcaceae produce acetate, which is then converted to butyrate by *Roseburia*. Butyrate is not only the main energy source of intestinal epithelial cells, but also an inhibitor of the signaling pathway of inflammatory cytokines (Nogal et al., 2021). *In vivo* and *in vitro* studies demonstrated that *Roseburia intestinalis* exerts anti-inflammatory effects by reducing the expression of IL17 (Zhu et al., 2018).

In CRC mice of this study, the balance of intestinal microbiota was significantly damaged with decreased abundance of these predominant intestinal microbe. Furthermore, the decreased infiltration of CD8^+^ T cells together with down-regulation of IL10 and up-regulation of IL17, STAT3 in the intestine, create an intestinal microenvironment prone to inflammation and the occurrence and development of colorectal cancer. In CRC mice treatment with FMT, the abundance of these intestinal bacteria increased significantly, and the disorder of intestinal flora was alleviated. Augmentation of CD4^+^ T cells, CD8^+^ T cells and CD49b^+^ NK cells, as well as increased expressions of IFNg, IL10, CXCR4, and reduced expressions of IL17, STAT3 screened from gene and protein levels, jointly create a microenvironment conducive to inhibit the progress of colorectal cancer.

Conversely, CRC mice were predominated by some intestinal bacteria, such as *Akkermansia, Bacteroides*, the *E. coli* complex, *Azospirillum, Mucispirillum, Erysipelatoclostridium, Clostridium, Parabacteroides, Turicibacter, Paeniclostridium*, and genera of Rikenellaceae. *Akkermansia muciniphila* degrades intestinal mucin and destroys the barrier function of the intestinal mucosa, prompting continuous stimulation of immune cells and inducing intestinal inflammation (Patel et al., 2022). The destruction of the barrier increases the frequency of carcinogenic bacteria contacting with intestinal mucosa and hence promotes cancerous pathogenesis. Mucin degradants of *Akkermansia* modulate the host immune system through signals such as TNFa, IFNg and IL10 (Li F. et al., 2019). *Bacteroides* promote CRC incidence by damaging DNA structure of intestinal epithelial cells and activating the EGFR and Wnt/β-cantenin signaling pathways (Cao et al., 2014; Dossa et al., 2016). *Bacteroides* *fragilis* directs the development of Foxp3^+^ Treg cells and increases the suppressive capacity of Tregs (Round and Mazmanian, 2010). Reduced infiltration of F4/80^+^ macrophages by Emodin administration inhibits colitis associated intestinal tumorigenesis (Zhang et al., 2021). *Bacteroides* are also involved in the metabolism of intestinal bile acids, which then induce the TGR5 signaling pathway in macrophages, contributing to the initiation or aggravation of inflammation and down-regulation of apoptosis, hence creating the environment for the development of CRC (Jia et al., 2018). The *E. coli* complex promote cancer progression by stimulating the expressions of COX2 and PGE2 in macrophages (Mola et al., 2020). Persistent infections with adherent and invasive *E. coli* give rise to chronic peripheral inflammation (Schmitz et al., 2019). Altered abundance of the *E. coli* complex positively correlates with the expression of pro-inflammatory factor IL6 (Cattaneo et al., 2017). Additionally, during inflammatory conditions, decreased expression of IL12 is along with reduce microbial translocation in the gut and significantly correlated with changes in beta diversity of intestinal microbiota (O’Connor et al., 2018; Pernomian et al., 2020). In addition, up to 40% of patients with *Clostridium* infection suffer from CRC. In CRC patients, the acidic and hypoxic environment resulting from anaerobic glycolysis of the cancer cells promotes spore germination of *Clostridium septicum*, resulting in serious infection (Mohammadi et al., 2022). And with the CRC development, TGFb transforms into a growth stimulator and promotes malignant transformation rather than inhibiting the growth of normal intestinal epithelial cells (Guzinska-Ustymowicz and Kemona, 2005). Oxidative stress caused by *Clostridium perfringens* infection induces inflammation and severe pathological changes in the intestinal mucosa (Liu et al., 2018). It was reported that the relative abundance of *Turicibacter* correlates with pro-inflammatory cytokines IL1 (Grant et al., 2021). In CRC mice received FMT, the abundance of these microbe decreased significantly, contributing to reversal of intestinal microbial dysbiosis. Furthermore, the reduced accumulation of Foxp3^+^ Treg cells and F4/80^+^ macrophages, up-regulation of TNFa, IFNg, IL10, down-regulation of IL1, IL6, IL12, TGFb, and decreased release of superoxide, may explain the role of FMT in inhibiting intestinal inflammation and CRC progression.

A large amount of intestinal bacteria have been found to be involved in regulating intestinal microenvironment. However, the interaction and dynamic balance of intestinal microbiota but no single strain, is crucial to maintain the normal function of the intestine. Fecal microbiota transplantation is regarded as an effective method for the transfer of healthy intestinal microbiota. For this, the selection of “ideal” intestinal microbiota is the key step for successful FMT, as the composition and quality of intestinal microbiota for each individual may differ greatly and is influenced by numerous factors, such as diet, environment and heredity. Currently, washed microbiota transplantation and artificially cultivated formula flora are developed to be safer and more effective method for fecal microbiota transplantation (Wu X. et al., 2021). In addition, the frequency of transplantation is another key point for successful FMT. In the process of FMT, a partial bacteria may be detached due to their weak adhesion or colonization resistance, resulting in failure of the introduced bacteria colonization. Therefore, multiple transplantations may be beneficial for bacterial colonization. In this study, 10 weeks of transplantation ensured that intestinal microbiota were stably colonized. However, in consideration of the patient’s compliance, it is necessary to seek the minimum frequency and duration time for microbial colonization stably.

## 5. Conclusion

Altogether, the results presented here indicate that intestinal microbiota transplantation is effective in reversing intestinal microbial dysbiosis of CRC mice. The balance of intestinal flora alleviates the CRC progression though inhibiting intestinal inflammation and enhancing anti-cancer immune responses, which is mediated by immune cells and inflammatory cytokines. This provides valuable information to evaluate the interactions of intestinal microbiota with CRC and for applying FMT in clinical treatment.

## Data availability statement

The datasets presented in this study can be found in online repositories. The names of the repository/repositories and accession number(s) can be found in the article/supplementary material.

## Ethics statement

The animal study was reviewed and approved by Institutional Research Board of Harbin Medical University.

## Author contributions

XQM and SLL: conceptualization. HY, XXL, XH, BXC, XHZ, DQX, YW, and ZKG: experiments. SG, LY, and LCY: methodology and data collection. HY, XXL, and SLZ: data analysis. XQM, GRL, and SLL: funding acquisition. SLL: supervision, writing and editing. XQM and XXL: drafting the manuscript. All authors contributed to the article and approved the submitted version.

## Funding

This work was supported by the National Natural Science Foundation of China (NSFC81903631, NSFC81971910, NSFC81871623, and NSFC82020108022), Youth Innovation Fund of University in Hei longjiang (UNPYSCT-2018065), and Postdoctoral Research Startup Fund of Heilongjiang Province (LBH-Q15080).

## Conflict of interest

The authors declare that the research was conducted in the absence of any commercial or financial relationships that could be construed as a potential conflict of interest.

## Publisher’s note

All claims expressed in this article are solely those of the authors and do not necessarily represent those of their affiliated organizations, or those of the publisher, the editors and the reviewers. Any product that may be evaluated in this article, or claim that may be made by its manufacturer, is not guaranteed or endorsed by the publisher.
